# Effects of capsinoid ingestion on energy expenditure and lipid oxidation at rest and during exercise

**DOI:** 10.1186/1743-7075-7-65

**Published:** 2010-08-03

**Authors:** Andrea R Josse, Scott S Sherriffs, Andrew M Holwerda, Richard Andrews, Aaron W Staples, Stuart M Phillips

**Affiliations:** 1 Exercise Metabolism Research Group, Department of Kinesiology, McMaster University, 1280 Main Street West, Hamilton, Ontario, L8S 4K1, Canada

## Abstract

**Background:**

The thermogenic and metabolic properties of capsinoids appear to mimic those of the more pungent sister compound capsaicin. However, few data exist on how capsinoid ingestion affects energy expenditure in humans and no data exist on its interaction with exercise. We aimed to determine how ingestion of capsinoids affected energy expenditure, lipid oxidation and blood metabolites at rest and during moderate intensity exercise.

**Methods:**

Twelve healthy young men (age = 24.3 ± 3 yr, BMI = 25.5 ± 1.7 kg·m^-2^) were studied on two occasions in a double-blind design following ingestion of either placebo or 10 mg of purified capsinoids at rest, after 90 min of cycling at 55% VO_2 _peak, and for 30 min into recovery. Subjects ingested the capsules 30 min prior to exercise.

**Results:**

At rest, following ingestion of capsinoids, we observed increases in VO_2 _and plasma norepinephrine levels, and decreases in concentrations of serum free fatty acids, plasma glycerol and the respiratory exchange ratio (all P < 0.05). At exercise onset, we observed a blunted accumulation of blood lactate with capsinoid ingestion vs. placebo (P < 0.05). There were no other significant differences between the conditions during or post-exercise.

**Conclusion:**

The ingestion of 10 mg of capsinoids increased adrenergic activity, energy expenditure, and resulted in a shift in substrate utilization toward lipid at rest but had little effect during exercise or recovery. The changes we observed confirm previous data on the thermogenic and metabolic effects of capsinoids at rest and further promote its potential role as an adjunct weight loss aid, in addition to diet and exercise.

## Introduction

Capsinoids are non-pungent analogues of capsaicin derived from the CH-19 sweet pepper [1]. Ingestion of capsinoids have been shown in most studies [2-5] but not all [6] to increase resting oxygen consumption, body temperature, and lipid oxidation in humans. As such, capsinoids appear to act in a manner similar to that of many adrenergic system agonists [2-4]. A major difference, however, is that capsinoids are broken down in the intestinal tract to yield vanillyl alcohol and a fatty acid [7-9]. This implies that the mechanism by which capsinoids work is likely neurally mediated via the vanilloid receptor (TRPV1) and gastric and intestinal neurons which trigger a centrally-mediated adrenergic response [7]. Capsinoids and capsaicin affect metabolism and thermogenesis in similar ways, but there are relevant differences. For example, capsaicin, due to its pungency and thus its additional effects on nociceptors [7], has been shown to increase blood pressure and heart rate shortly after ingestion whereas capsinoids do not [2]. Thus, given the complete lack of pungency, the use of capsinoids may represent a more viable longer-term adjunct therapy for the treatment of overweight and obesity as it has been shown to be effective in preventing weight gain in humans [3,4].

Obesity-related programs that do not involve pharmacological interventions advocate the use of diet and/or exercise as treatment [10-13]. One of the biggest detractions of diet-induced weight loss is a reduction in resting metabolic rate [11,12], which has been counteracted by the use of adrenergic drugs [14-16]. These drugs increase metabolism; however, their side-effect profiles do not recommend them for widespread use [17,18]. Despite this health risk, it has been well documented that thermogenic agents are used with great frequency by people attempting to lose weight [19] and can even be done so effectively [20]; however, a lower side-effect burden would likely be beneficial.

Capsinoids are non-pungent thermogenic compounds that promote lipid oxidation, and as mentioned have a very favourable toxicity and side-effect profile [1]. Thus, capsinoids could be used to support diet and/or exercise-induced weight loss in the treatment of overweight and obesity [2-5]. With this in mind, it would be important to test this compound in a clinical setting to determine its efficacy as an adjunct weight loss aid. As a preliminary step, we set out to test capsinoids in conjunction with exercise to see if the thermogenic and metabolic effects at rest are maintained or even potentiated with exercise. Thus, the purpose of this study was to examine the response of young healthy males to a 90 min bout of moderate intensity endurance exercise after having consumed 0 mg (placebo) or 10 mg of capsinoids. We proposed that capsinoid ingestion 30 min prior to exercise would generally induce metabolic changes consistent with increased metabolic rate and lipid oxidation, namely: elevate resting oxygen consumption; a shift in metabolism (respiratory exchange ratio [RER]) towards greater lipid oxidation; and increase catecholamine release consistent with a stimulation of the adrenergic system. We further hypothesized that the aforementioned changes would be maintained with moderate exercise.

## Methods

### Subjects

Young healthy males between the ages of 18 and 30 years were recruited from McMaster University and the surrounding Hamilton area. All participants were screened prior to inclusion for standard medical conditions that would preclude their participation in the trial. Subjects were deemed healthy based on their responses to the medical screening questionnaire and thus were eligible to participate. Subjects were also required to be recreationally active defined as exercising at least 2 times, but no more than 5 times per week, and have a peak VO_2 _of > 40 ml/kg/min as determined by a maximal progressive exercise test. Exclusion criteria included smoking and the use of interfering medications, natural health products or dietary supplements.

Subjects were informed of the potential risks and procedures associated with the study and gave their written informed consent prior to participation. The protocol was approved by the Research Ethics Board at McMaster University and Hamilton Health Sciences and conformed to all standards of Canada's Interagency Panel on Research Ethics for conducting human research http://www.pre.ethics.gc.ca/eng/index/. The capsinoids also underwent review by Health Canada's Natural Health Products Directorate (NHPD) and a notice of authorization was obtained (NHPD # 130269) before ethics approval was granted.

### Test substance

Capsules contained an extract from the pepper fruit variety CH-19 Sweet. (*Capsicum anuum *L.). Capsinoid oil was extracted as follows: the dried fruit was treated with hexane, and fruit sediment was removed by filtration, followed by evaporation and distillation with medium-chain triacylglycerol and column chromatography to yield purified capsinoids. Capsinoids consisted of capsiate, dihydrocapsiate and norhydrocapsiate in a 70:23:7 ratio (as determined by High Performance Liquid Chromatography [HPLC]). The purified capsinoids were then dissolved in rapeseed oil and encapsulated in vegetarian softgel capsules made of modified maizestarch, vegetable glycerine and carageenan; each capsule contained 1 mg of capsinoids and 199 mg of a mixture of rapeseed oil and medium-chain triacylglycerols. All capsules were manufactured in one batch. Stability tests determined that the product would be stable beyond the duration of the trial.

### Protocol

#### Conditions

We used a double-blind, placebo-controlled, repeated measures study design in which each subject completed 2 trials; 0 mg and 10 mg of capsinoids in random order. Based on the acute results of previous studies [2,4], we anticipated that a sample size of 12 subjects (all male; to control for possible sex-based differences in substrate oxidation during exercise) would be adequate to see significant effects of capsinoids on oxygen consumption and substrate oxidation. Capsinoids were ingested in capsule form. All capsules looked identical and contained either 1 mg of capsinoids or not (rapeseed oil only). Ten small capsules were consumed at each trial. Subjects were instructed to consume the capsules in 1 minute when given and were allowed water throughout the trial *ad libitim*. McMaster University Pharmacy controlled the randomization and the code for each treatment was given to the principal investigator only after completion of the data analysis.

#### Pre-trial testing

##### Peak VO_2 _test

Once informed consent was obtained, subjects were asked to come to the Exercise Metabolism Research Group (EMRG) laboratory at McMaster University to establish their peak VO_2 _using an incremental ramp protocol on a braked cycle ergometer (Lode, Groningen, The Netherlands). Subjects rode at 75W for 2 min as a warm-up after which the workload was progressively increased 0.5 W/s until the subject could no longer continue. Criteria for stopping the VO_2 _peak test included, a) failure to maintain a cadence of 60 rpm; b) reached within 10% of calculated maximal heart rate (220 - age); c) had an RER > 1.15. Failure to meet these criteria meant the subject did not reach their peak oxygen consumption and was required to repeat the test. All 12 subjects achieved their peak VO_2 _in one test.

##### Familiarization ride

Once the subject's peak VO_2 _was established, they were asked to return to the EMRG laboratory for an additional pre-trial ride where we aimed to verify the workload corresponding to 55% of each subject's peak VO_2_. This exercise intensity is adequate to elevate lipolysis (i.e., increase circulating glycerol and FFA), catecholamines, and show a substantial shift in RER toward lipid oxidation with increasing exercise duration [21-23]. Moreover, this type of exercise intensity represented an intensity that elicited an exercising RER at or around 0.85, a level at which a good blend of fuels are being oxidized, but is also an intensity close to what has been referred to as 'fatmax', which is defined as the intensity of exercise at which the maximal *rate *of lipid oxidation occurs [19,24-26]. Before the test, we calculated this workload according to the algorithm detailed by Latin et al. [27], and had subjects pedal just below (-10 W), at, and just above (+10 W) the calculated workload while measuring VO_2_, VE and HR during the ride. This ride took ~30-40 minutes to complete; 10 min at each workload. A successful test occurred when the workload that elicited a VO_2 _of 55 ± 1% of the measured peak VO_2 _was achieved. The workload established during this test was then to be the workload the subject pedaled at during the trials. Following the successful ride, we recorded the seat and handle bar settings which felt most comfortable in order to have the bike ready for the subject on subsequent trial days.

#### Testing

##### Trial days

Subjects reported to the lab twice in the morning for their trials at either 0700 h, 0900 h or 1100 h. Exercise trials took place after an overnight fast of 10-12 hours. Subjects recorded their evening meal the night before the first trial, and in order to standardize pre-trial conditions, subjects were asked to consume the same meal the night before the next trial. Having subjects in the fasted state does not present a risk or barrier to the subjects completing the trial as based on past experience with this protocol [21,23,28]. Moreover, being in the fasted state during exercise is, from a mechanistic standpoint, the easiest state in which to interpret the data on substrate oxidation. Subjects were also asked to refrain from consuming alcohol and caffeine the night before (from 1800 h onwards) the trial. Trials were separated by 1 week, thus each subject was to maintain the same trial time and day of the week for 2 weeks in order to complete the study.

**Figure 1:**
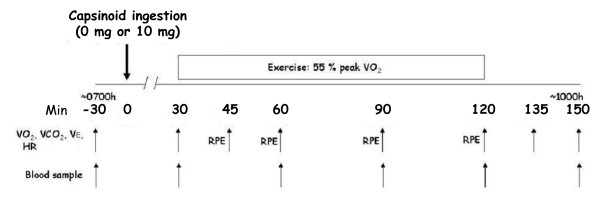
**Schematic representation of the protocol to test the impact of capsinoids on resting and exercising energy expenditure**. VO_2 _- oxygen consumption, VCO_2 _- carbon dioxide production, VE - ventilation, HR- heart rate and RPE - rate of perceived exertion (according to Borg's scale). Capsinoids/placebo ingestion occurred at 0 min.

Upon arrival to the lab, subjects were asked to sit and relax (~30 min) to become acclimatized to the room before the first blood sample was taken. Also during this time, subject's weight was taken and several questions pertaining to sleeping patterns, food consumption and medication use from the previous day were answered. The trial then proceeded according to the schematic timeline shown in **Figure **1. Ambient temperature in the lab was 22 ± 1ºC and relative humidity was always less than 50%. Baseline heart rate (HR), metabolic cart measures (VO_2_, VCO_2_, RER, VE) and a blood sample were then taken. After 30 minutes (noted as time 0 min; **Figure **1), subjects' ingested 10 small (1 mg each) gel capsules according to their respective randomization allocation (0 mg or 10 mg of capsinoids) with water *ad libitim*. At 30 minutes post-ingestion (60 minutes into protocol), subjects started cycling. They rode for 90 minutes at 55% of their peak VO_2 _(as previously determined) and recovered for 30 minutes. HR, blood samples and metabolic cart measurements were taken at several time points during the ride and into recovery (Figure 1). The whole trial lasted 180 minutes.

### Analyses

#### Expired Gas Analysis

We collected expired gas samples (breath-by-breath and ensemble averaged into 30s bins) using a metabolic cart (AEI Technologies, Moxus respiratory gas analyzer, Pittsburgh, PA) at several time points during and also 30 min into recovery to assess ventilation (VE), oxygen uptake (VO_2_), carbon dioxide production (VCO_2_) and respiratory exchange ratio (RER).

#### Blood Metabolites

Blood samples were taken in two *Vacutainer *tubes per time point, one with sodium heparin and the other with no additives to obtain plasma and serum, respectively. Samples were subsequently processed and stored in -20°C freezers for later analysis. Whole blood was also immediately analyzed for blood glucose (Accu-Check, Roche Diagnostics Canada, Laval, PQ) and blood lactate (Accu-Trend, Roche Diagnostics Canada, Laval, PQ). Upon study completion, samples were then analysed for various metabolites. All samples were analyzed in duplicate and inter-sample coefficients of variation never exceeded 5%. Plasma was analyzed for glycerol using a fluorometric assay system based on the procedures outlined in detail elsewhere [21,22]. Serum free fatty acids were analyzed using a commercially available kit (HR Series NEFA-HR(2), Wako Diagnostics, Richmond, VA). Plasma treated with glutathione was analyzed for concentrations of epinephrine and norepinephrine using high performance liquid chromatography (HPLC) analysis as described previously [21].

### Rate of Perceived Exertion

We measured subject's perceived exertion during the exercise with a validated visual analogue scale (Borg Scale).

### Statistics

All data were analyzed using a repeated measures two-way analysis of variance (ANOVA) with time as a within factor and dose as a between factor. Significant ANOVA effects were further analyzed using Tukey's test as a post-hoc procedure; P < 0.05 was deemed to be significant. Changes in resting values from pre- to post-capsiate/placebo ingestion were compared using a paired t-test. Values are presented means ± SD, n = 12 per data point unless otherwise noted.

## Results

### Subject characteristics

Twelve male subjects participated in this study. Subject characteristics are shown in **Table **1. Subjects were all relatively fit with a mean peak VO_2 _of 43.3 ± 2.1 ml/kg/min.

**Table 1:** Subject characteristics

Age (yr)	24 ± 3
Weight (kg)	83.0 ± 10.5
Height (m)	1.80 ± 0.08
Body Mass Index (kg^.^m^-2^)	25.5 ± 1.7
Peak VO_2 _(ml/kg/min)	43.3 ± 2.1
Peak VO_2 _(l/min)	3.58 ± 0.44

### Oxygen Consumption and Respiratory Exchange Ratio (VO_2 _and RER)

**Figure 2:**
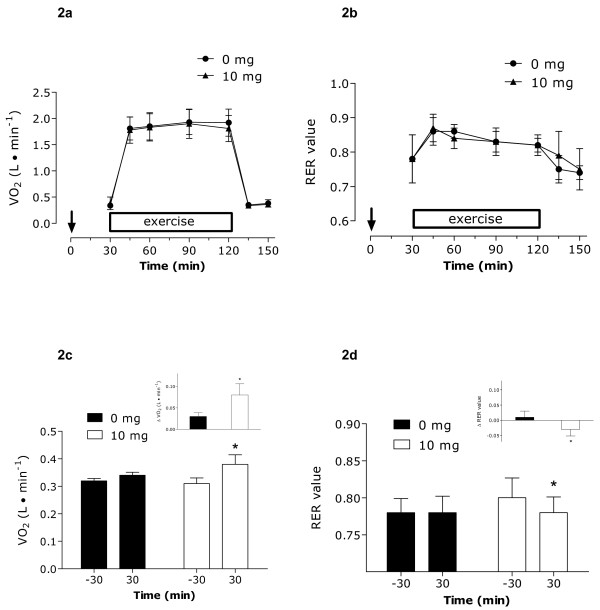
**Oxygen uptake during the protocol from the time of ingestion**. **(a)**. Respiratory exchange ratio (RER) during the protocol from the time of ingestion (b). Oxygen uptake at rest only; measured at -30 min and 30 min (c). Inset c represents the change in resting VO_2_. RER at rest only measured at -30 min and 30 min (d). Inset d represents the change in resting RER. * Significantly different (P < 0.05) from the same value at rest or than the other condition (inset). ↓ Ingestion of capsinoids/placebo at 0 min. Values are means ± SD (n = 12).

No differences were observed between treatments for VO_2 _or RER during the trial (**Figures **2a and 2b). Examination of the resting values only (-30 to 30 min of protocol), we observed that resting VO_2 _was significantly higher and resting RER was significantly lower following ingestion of 10 mg of capsinoids versus baseline and 0 mg (**Figures **2c and 2d). When expressing the metabolic rate (l/min) as kcal/min, the resulting resting values pre and post ingestion were: placebo at rest: 1.54 kcal/min to 1.64 kcal/min; capsinoid at rest: 1.49 kcal/min to 1.83 kcal/min.

### Heart Rate (HR) and Rating of Perceived Exertion (RPE)

We observed the expected rise in exercise HR in all subjects, but did not observe any differences between trials at rest or during exercise. Similarly, we did not see any differences between trials in subjects' rating of perceived exertion (data not shown).

### Blood Metabolites

**Figure 3:**
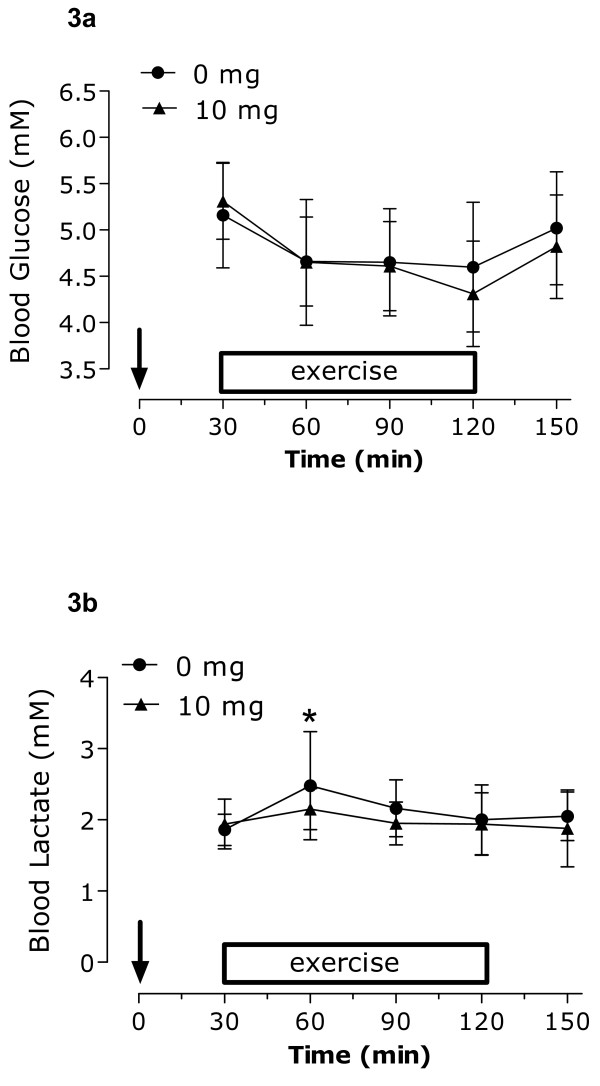
**Blood glucose (a) and lactate (b) concentrations during the protocol from the time of ingestion**. *Significantly different (P < 0.05) from rest (30 min) in the placebo group only. ↓ Ingestion of capsinoids/placebo at 0 min. Values are means ± SD (n = 12).

**Figure 4:**
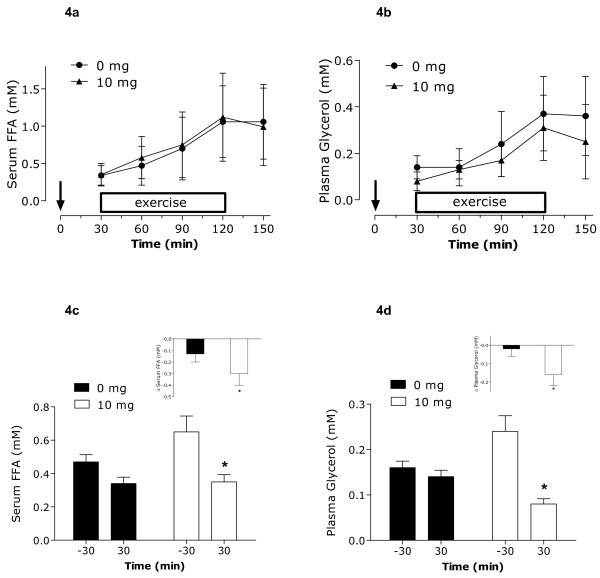
**Serum free fatty acids (FFA; a) and plasma glycerol (b) concentrations during the protocol from the time of ingestion**. Serum FFA at rest only; measured at -30 min and 30 min (c). Inset c represents the change in resting FFA concentration. Plasma Glycerol at rest only; measured at -30 min and 30 min (d). Inset d represents the change in resting glycerol concentration. * Significantly different (P < 0.05) from the same value at rest or than the other condition (inset). ↓ Ingestion of capsinoids/placebo at 0 min. Values are means ± SD (n = 12).

**Figure 5:**
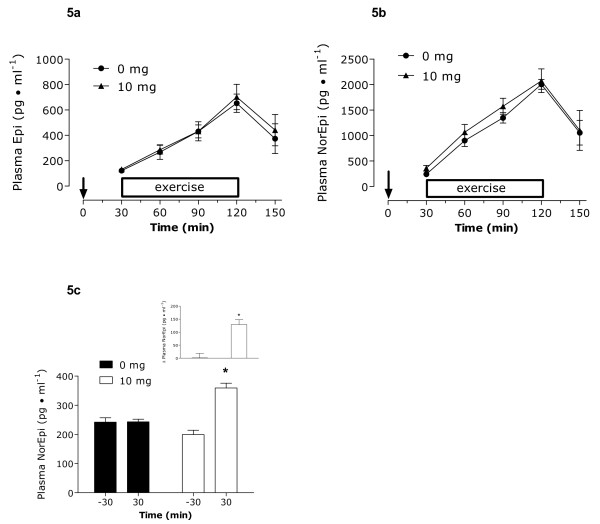
**Plasma Epinephrine (Epi; a) and norepinephrine (Norepi; b) concentrations during the protocol from the time of ingestion**. Norepinephrine concentrations at rest only; measured at -30 min and 30 min (c). Inset c represents the change in resting norepinephrine. * Significantly different (P < 0.05) from the same value at rest or than the other condition (inset). ↓ Ingestion of capsinoids/placebo at 0 min. Values are means ± SD (n = 12).

Blood glucose remained stable and unchanged throughout all phases of the protocol, including rest, with no significant difference between conditions (**Figure **3a). At rest and during the last hour of the protocol, no differences between groups were observed in blood lactate concentrations. There was no observed rise in lactate levels during the trial following the consumption of capsinoids. Thirty minutes into exercise (at 60 min), blood lactate levels were significantly elevated compared to rest (30 min) in the placebo group only (**Figure **3b). Concentrations of serum FFAs and plasma glycerol increased in both trials to a similar extent after the onset of exercise (**Figures **4a and 4b). Upon examination of the resting values only (-30 to 30 min of protocol), serum FFAs and plasma glycerol were significantly reduced at 30 min compared to starting levels (-30 min) following ingestion of 10 mg of capsinoids only. No changes were observed in the placebo group (**Figures **4c and 4d). When expressing fat oxidation as kcal/min (indirectly using RER and VO_2_), the resulting resting values pre and post ingestion were: placebo at rest: 1.18 kcal/min to 1.26 kcal/min; capsinoid at rest: 1.19 kcal/min to 1.41 kcal/min. The responses of plasma norepinephrine and epinephrine are show in **Figures **5a and 5b, respectively. Overall, there were no differences between trials in the adrenergic hormones. When looking only at the resting time points (-30 min and 30 min), plasma norepinephrine concentrations were significantly higher at 30 min compared to -30 min following ingestion of 10 mg of capsinoids but not placebo (**Figure **5c). There was no change in plasma epinephrine at rest.

### Hydration

The average *ad libitim *water intake during the trial was ~1L (2 × 500 ml water bottles) with no differences between groups. No subject reported feeling any different (i.e. increased HR, sweating, breathing rate, etc.) between the trials.

## Discussion

The main findings of this investigation were that, versus a placebo, 10 mg of capsinoids induced a rise in resting oxygen consumption, a decline in RER, an increase in plasma norepinephrine, and a decline in serum FFAs at rest. We also observed that 10 mg of capsinoids blunted the small but significant rise in blood lactate observed at the onset of exercise seen in the placebo group (0 mg).

Our original hypothesis was that we would see the aforementioned changes in VO_2_, RER and catecholamines at rest, in line with previous studies [2-4], and that these differences would carry over and be maintained or even augmented during exercise. This type of response has been observed with another adrenergic agonist, caffeine [29]. We did not observe this response and, in fact, aside from the slightly lower blood lactate seen with capsinoid ingestion there was no effect at rest that carried over into exercise. Thus, we propose that the usual adrenergic response associated with exercise may have been greater than that which was induced by capsinoids at rest thereby overriding any significant differences seen during the exercise phase. Hence, there was no synergistic or additive effect at this intensity of exercise. **Figure **5 shows that the increases seen in plasma epinephrine and norepinephrine during exercise far exceed the magnitude of increase seen at rest with 10 mg of capsinoids. Therefore, it is not surprising that these subtle differences between treatments disappeared with exercise. Our findings do, however, further support claims that capsinoids have their greatest thermogenic effect at rest.

The consumption of 10 mg of capsinoids resulted in a rise in plasma norepinephrine and a decline in RER. Both of these findings suggest that capsinoids increased whole-body lipid oxidation at rest. Furthermore, both plasma glycerol and serum FFA concentration decreased concomitantly possibly indicating the body's increased reliance on circulating lipids as fuel at that time; without tracer-based estimates of appearance and disappearance we are unable to confirm our thesis. Similar findings with respect to lipid metabolism were reported in a longer term study by Kawabata et al [3]. Researchers fed human subjects CH-19 sweet peppers (0.13 g/kg before each meal [0.4 g/kg total]) for 2 weeks and controlled their diet. Those who consumed capsinoids showed a decrease in visceral fat (assessed by CT scan), a decrease in resting RER, and a significantly greater weight loss compared to the control group. Moreover, there was a significant correlation between weight lost and SNS activity (R^2 ^= 0.66; P < 0.05) [3]. In a more recent study, 80 overweight men and women were fed 6 mg/d of capsinoids or placebo for 12 weeks. While total body weight and % body fat did not differ between groups, the capsinoid group showed reductions in visceral fat assessed by DXA and increases in fat oxidation assessed by indirect calorimetry [5]. While these studies certainly show evidence for capsinoids being adrenergic system stimulants and activating lipid oxidation, they do little to shed light on a potential mechanism. As previously mentioned, tracer-based measures are required to assess the mechanistic underpinnings responsible for the decline in RER and increased lipid oxidation and visceral fat loss with capsinoids. With respect to capsinoids' positive effect on the sympathetic nervous system (SNS), this phenomenon has been demonstrated consistently in other human studies [2-4].

We also observed an increase in metabolic rate as indicated by the increase in resting VO_2 _with the consumption of capsinoids. Our findings are similar to most [2,4] but not all [6] of those reported previously in humans showing increased oxygen consumption and heat production indicative of SNS activation. Moreover, as also reported previously [2], we demonstrated that capsinoids did not affect resting heart rate measures despite the observed changes in plasma norepinephrine and energy expenditure. In a study by Ohnuki et al. [4], increases in resting oxygen consumption were reported to be in the range of ~5% with consumption of 0.1 g/kg of CH-19 sweet peppers (i.e. for a 70 kg male, this corresponds to 7 g of CH-19 sweet peppers and ~5 mg purified capsinoids [0.3-1.0 mg capsinoids/g pepper [4]]). We report here that 10 mg of purified capsinoids extracted from CH-19 sweet peppers, a much larger dose, induced a significant rise in resting oxygen consumption of a little more than 20% or 70 ± 13 ml/min. Therefore, the increase in resting VO_2 _observed in our study, although greater in magnitude possibly due to the larger dose, seems to be in line with other published research [2,4]. Although we did not measure core body temperature in the current study, with a dose of 0.1 g/kg of CH-19 sweet peppers, researchers observed a significant increase in core body temperature 10-60 min post ingestion further supporting an increase in thermogenesis with capsinoids [4].

This study was the first to assess the effect of capsinoids and aerobic exercise together in humans on measures of substrate oxidation and energy expenditure. None of the observed resting differences between groups were maintained with exercise. However, at the onset of exercise, the ingestion of 10 mg of capsinoids blunted the rise in blood lactate observed in the placebo group, although significant, this effect was quite subtle. A number of mechanisms are thought to affect lactate production and clearance during exercise, especially at the onset. These include oxygen availability, pyruvate dehydrogenase activation, lactate dehydrogenase isozymes, as well as lactate transporter content [30]. While we cannot be entirely sure as to why lactate did not increase to the same extent in both groups at the start of exercise, we speculate that it may be due to an increased reliance by the 10 mg capsinoids group on available FFAs as fuel early in exercise as opposed to glycogen. Although quite plausible, further studies would need to confirm this hypothesis.

The results of the current study lead us to believe that capsinoids' effects on resting metabolism do not carry over into exercise. It is entirely possible, however, that our exercise regimen may have been too intense and that a lower intensity exercise such as a fast-paced walk would have allowed us to see maintenance of some of the resting metabolic and thermogenic effects. Further studies are needed to assess this, possibly implementing an exercise regimen that more closely mimics daily routines of the general North American population, i.e. activity that is less strenuous and shorter. It would also be interesting to test the effect of capsinoids with a 45-60 minute brisk walk and to monitor for 1 hour post-exercise to allow for a more complete metabolic recovery. Moreover, a test of the effect of capsinoids under these conditions in overweight and obese, sedentary persons may reveal effects during exercise not seen in fit, normal weight individuals.

In conclusion, 10 mg of purified capsinoids resulted in increased thermogenesis following ingestion in young, healthy, physically active males. In this crossover, double-blind, acute trial, compared to a placebo, capsinoids increased resting energy expenditure by ~20%, and based on several observations relating to lipid metabolism (decreased RER, decreased levels of FFAs and glycerol in blood, increased SNS activity), capsinoid ingestion induced greater lipid oxidation at rest. Although none of these effects carried over into exercise, the blunted lactate response with capsinoids 30 min into the exercise bout suggests an altered substrate use, namely a greater reliance on fat as fuel, at the start of exercise. This study, in conjunction with the current literature, further proves that the subtle metabolic effects of capsinoids are most pronounced at rest and as demonstrated in this trial, they seem to be superseded by a moderate exercise regimen.

## Competing interests

This work was supported by a grant from Ajinomoto Inc. The funder had no say in the study design, the interpretation of data, nor in the contents of the final manuscript.

The authors declare that they have no competing interests, financial or otherwise.

## Authors' contributions

SMP acquired the research grant. SMP and ARJ participated in the design of the study. ARJ, SSS and RA carried out the study. AMH and AWS conducted laboratory analyses. ARJ and SMP performed the statistical analyses wrote the manuscript. All authors read and approved the final manuscript.
